# Effects of physical activity on cardiorespiratory fitness and vascular function in adults aged 50–60 years

**DOI:** 10.3389/fpubh.2026.1800039

**Published:** 2026-03-25

**Authors:** Yingying Diao, Wenqing Yan, Xinlei Han, Yifei Zhang, Meihua Su, Jianming Chen

**Affiliations:** 1 College of Physical Education, Jimei University, Xiamen, Fujian, China; 2 School of Science, Jimei University, Xiamen, Fujian, China; 3 School of Foreign Languages, Jimei University, Xiamen, Fujian, China

**Keywords:** cardiorespiratory fitness, exercise, physical activity, vascular function, VO₂max

## Abstract

**Objective:**

To examine the associations between physical activity and cardiorespiratory fitness and vascular function in adults aged 50–60 years, and to investigate the moderating role of cardiorespiratory fitness in the relationship between physical activity and vascular function.

**Methods:**

This cross-sectional study included 155 non-manual workers aged 50–60 years. Physical activity was assessed using the International Physical Activity Questionnaire–Short Form (IPAQ-SF). Cardiorespiratory fitness and vascular function were evaluated using established physiological indicators.

**Results:**

Physical activity was significantly associated with both cardiorespiratory fitness and vascular function, with evident sex differences. In men, cardiorespiratory fitness increased progressively with higher physical activity levels, whereas in women, significant improvements were observed only at high physical activity levels. Vascular elasticity appeared to be more responsive to physical activity in women. Cardiorespiratory fitness significantly moderated the association between physical activity and vascular function, with a stronger moderating effect observed in men. The beneficial effect of physical activity on vascular function was most pronounced among individuals with lower levels of cardiorespiratory fitness.

**Conclusion:**

Among adults aged 50–60 years, physical activity is positively associated with cardiorespiratory fitness and vascular function and exerts beneficial effects on vascular health, with notable sex differences. Furthermore, cardiorespiratory fitness moderates the relationship between physical activity and vascular function, suggesting that the vascular benefits of physical activity vary according to sex and individual fitness levels.

## 1 Introduction

Cardiovascular disease (CVD) represents a major threat to human health and has become one of the most pressing global public health challenges. CVD accounts for nearly half of the global burden of non-communicable diseases and remains the leading cause of mortality worldwide. In 2019, approximately 18.6 million deaths were attributed to CVD, a number projected to rise to 23.6 million by 2030. This trend is closely linked to global population aging: the number of individuals aged ≥65 years is expected to reach 1 billion by 2050, and in developed countries, older adults already account for nearly three-quarters of CVD-related deaths (1).

In this context, adults aged 50–60 years represent a critical window for CVD prevention and control. The population size of this age group continues to expand, and increased life expectancy allows prolonged accumulation of major risk factors such as hypertension and diabetes, rendering this period particularly suitable for early intervention prior to the onset of clinical events. Effective prevention strategies targeting this population are therefore essential to curb the growing burden of CVD and to alleviate future health and economic pressures, especially in low- and middle-income countries.

In China, both the prevalence and mortality of CVD remain on an upward trajectory. According to the *Report on Cardiovascular Health and Diseases in China 2020* (2), the estimated number of individuals with CVD has reached approximately 330 million, and CVD-related mortality ranks first among all causes of death, posing a serious threat to population health. Beyond its detrimental effects on physical and mental well-being, CVD also imposes substantial economic burdens on families and society, highlighting the urgency of effective preventive and therapeutic measures.

Evidence suggests that impairment of large artery structure and function constitutes an early pathological process, while advanced vascular dysfunction is a major risk factor for the development and progression of CVD (3). Early detection of abnormalities in arterial structure and function, followed by timely intervention, can effectively delay CVD onset and progression. Accordingly, early prevention of vascular dysfunction represents a fundamental strategy for CVD prevention.

Physical activity, as an efficient, economical, and safe non-pharmacological intervention, has been widely recognized for its role in CVD prevention and management. A meta-analysis including over 80,000 participants demonstrated that regular and moderate physical activity was associated with a 33% reduction in all-cause mortality and a 35% reduction in CVD-related mortality (4). Furthermore, a large prospective cohort study with a 34-year follow-up reported that long-term engagement in physical activity extended life expectancy by 7–8 years and substantially reduced CVD mortality risk (5).

The health benefits of physical activity exhibit a dose–response relationship. Within a certain range, increasing exercise intensity and volume may lead to progressively greater health benefits or reach a plateau effect (6). Therefore, this study focuses on vascular health and cardiorespiratory fitness to examine the associations between physical activity and vascular and cardiorespiratory function among adults aged 50–60 years, with the aim of providing scientific evidence for the development of exercise prescriptions targeting vascular stiffness.

## 2 Materials and methods

### 2.1 Study design and sample size calculation

This study adopted a cross-sectional design. Based on previous research (7), a two-sided test was applied with a significance level of *α* = 0.05 (confidence level = 95%) and an assumed standard deviation of 30. Sample size estimation was conducted using SPSS version 26.0, yielding a minimum required sample size of 126 participants. Considering a potential 20% rate of invalid questionnaires or missing data, at least 150 participants were required for inclusion.

The study protocol was reviewed and approved by the Ethics Committee of Jimei University (Approval No. 20260111001). All participants provided written informed consent prior to participation, in accordance with the Declaration of Helsinki.

### 2.2 Participants

Participants were recruited through both online and offline methods. Eligible participants were non-manual workers aged 50–60 years.

Inclusion criteria were as follows:

(1) Aged between 50 and 60 years;
(2) Non-manual occupation;
(3) No history of sports-related injuries;
(4) No absolute contraindications to exercise;
(5) No cognitive impairment;
(6) No mobility limitations;
(7) No malnutrition;
(8) No chronic sleep deprivation;
(9) No diagnosed psychological disorders.

Exclusion criteria included:

(1) Presence of sports injuries;
(2) Implanted metal devices such as pacemakers or coronary stents;
(3) Diseases with absolute contraindications to exercise;
(4) Mobility impairment or cognitive abnormalities;
(5) Invalid or randomly completed Physical Activity Readiness Questionnaire (PAR-Q);
(6) Inability to complete the testing procedures.

Participants meeting any of the above exclusion criteria were not included in the final analysis.

### 2.3 Assessment of demographic characteristics and physical activity

Sociodemographic information was collected using a self-administered questionnaire. Alcohol consumption was coded as follows: never drinking = 0, social drinking = 1, and heavy drinking = 2. Social drinking was defined as alcohol intake ≤25 g/day for men and ≤15 g/day for women; intake exceeding these thresholds was classified as heavy drinking (8).

Physical activity was assessed using the short form of the International Physical Activity Questionnaire (IPAQ-SF), which captures physical activity intensity, duration, frequency, and type over the previous 7 days. The IPAQ-SF has demonstrated good reliability and validity in Asian populations (9, 10).

Different types of physical activity were converted into metabolic equivalents of task (METs) to quantify total physical activity. Based on established criteria, participants were categorized into low (L), moderate (M), and high (H) physical activity groups (11, 12). The classification criteria are presented in Table 1. Total physical activity volume was calculated according to the *IPAQ Scoring Protocol* (13) using the following formula:

**Table 1:** Classification criteria for total physical activity (31).

Level of physical activity	Physical activity level
Low levels of physical activity	Total weekly walking or moderate-to-vigorous physical activity for 5 or more days <600 MET-minutes/week
Moderate level of physical activity	Total weekly walking or moderate-to-vigorous physical activity for 5 days or more, with a total of 600 ≤ *X* ≤ 3,000 MET-minutes/week
High level of physical activity	Total weekly walking or moderate-to-high-intensity physical activity for 5 days or more, with a total of ≥3,000 MET-minutes/week

### 2.4 Clinical and physiological measurements

#### 2.4.1 Anthropometric measurements

Height was measured using a standard stadiometer. Waist-to-hip ratio (WHR) was assessed using a flexible tape measure, with waist circumference measured at the level of the umbilicus and hip circumference measured at the level of the pubic symphysis. WHR was calculated as waist circumference divided by hip circumference.

Body composition was assessed using a bioelectrical impedance analyzer (InBody 770, Korea). Participants were instructed to wear light clothing and stand barefoot on the device, with both feet placed on the electrodes and both hands gripping the handles, maintaining a stable posture throughout the measurement.

#### 2.4.2 Cardiorespiratory fitness

Maximal oxygen uptake (VO₂max) is considered one of the gold-standard indicators of cardiorespiratory fitness. Given the safety concerns associated with direct maximal testing, VO₂max was estimated indirectly using the Astrand–Rhyming protocol. Prior to testing, participants wore a heart rate monitor and underwent pre-exercise blood pressure and electrocardiogram screening to ensure eligibility.

VO₂max testing was conducted using a cycle ergometer (ERGOSANA XC-1000, Germany). Exercise workload was selected based on participants’ age, sex, and exercise background (14), and progressively increased during a 6-min cycling protocol. Heart rate was recorded at the end of the 5th and 6th minutes. Absolute VO₂max values were estimated using the Astrand–Rhyming nomogram, followed by correction for age, height, and body mass to obtain relative VO₂max (mL·kg^−1^·min^−1^) (15). Relative VO₂max was used as the indicator of cardiorespiratory fitness in the moderation analysis.

#### 2.4.3 Vascular function

Cardio-ankle vascular index (CAVI) and ankle–brachial index (ABI) were used as indicators of vascular function. CAVI reflects arterial stiffness, with lower values indicating greater arterial elasticity, while ABI reflects lower-limb blood pressure and vascular status, with higher values indicating better vascular health. Both indices are established predictors of CVD risk (16).

The CAVI and ABI were measured bilaterally using an automated vascular screening device (Omron BP-203RPEIII, Japan), following the manufacturer’s standardized protocol. Participants rested in a supine position while limb cuffs were applied to all four extremities. A phonocardiographic microphone was placed at the cardiac apex (left fifth intercostal space, 1–2 cm medial to the midclavicular line). Participants were instructed to remain still and quiet during the measurement. Testing commenced once stable heart sounds and sinus rhythm were observed on electrocardiography.

### 2.5 Statistical analysis

All statistical tests were two-tailed, and statistical significance was set at *p* < 0.05. Data preprocessing and statistical analyses were conducted using SPSS version 26.0 (IBM Corp., Armonk, NY, United States). Six outliers were excluded, resulting in a final sample of 155 participants for subsequent analyses. The normality of continuous variables was assessed using the Shapiro–Wilk test.

Continuous variables are presented as mean ± standard deviation (SD), and categorical variables are expressed as frequency and percentage [*n* (%)]. Differences in anthropometric indices among different physical activity levels were compared using one-way analysis of variance (ANOVA). When a significant overall *F*-value was observed, *post hoc* pairwise comparisons were performed using the Least Significant Difference (LSD) test.

To examine the associations between physical activity, cardiorespiratory fitness, and vascular function, analyses were first conducted in the overall sample and further stratified by sex. The pooled analyses were used to evaluate population-level associations, whereas sex-stratified analyses were performed to assess potential effect modification by sex. These subgroup analyses were prespecified based on prior evidence suggesting sex-specific physiological responses to exercise.

Pearson correlation analysis was conducted to examine the relationships between physical activity, cardiorespiratory fitness, and vascular function. Moderation analysis was performed using Hayes’ PROCESS macro (Model 1) for SPSS. After controlling for the main effects, the interaction term between physical activity and cardiorespiratory fitness was entered into the regression model to assess its effect on vascular function. Simple slope analysis was subsequently conducted to further characterize the nature of the moderation effect.

All statistical tests were two-tailed, and statistical significance was set at *p* < 0.05.

### 2.6 Quality control

All participants were recruited strictly according to the predefined inclusion and exclusion criteria. Questionnaires with random or inconsistent responses and participants who failed to cooperate with testing procedures were excluded.

All data collectors received standardized training and were familiar with questionnaire administration, instrument operation, and testing protocols to minimize measurement bias. Prior to data collection, all devices underwent standardized calibration and quality control procedures. Laboratory equipment was inspected and maintained by professional technicians and engineers to reduce measurement error.

Data entry was performed independently by two researchers, followed by cross-checking to ensure accuracy. Statistical analyses were conducted independently by a third researcher.

## 3 Results

### 3.1 Baseline characteristics of participants by physical activity level

A total of 155 participants were included in the final analysis, comprising 74 men (47.74%) and 81 women (52.26%), with a mean age of 54 years. Based on physical activity classification using the IPAQ-SF, 48 participants (30.92%) were categorized into the low physical activity group (L), 58 participants (36.18%) into the moderate physical activity group (M), and 49 participants (32.89%) into the high physical activity group (H).

Based on questionnaire data, participants in the low physical activity group primarily engaged in sedentary behaviors, limited walking, or light household activities. The moderate physical activity group mainly participated in activities such as aerobic exercise, Tai Chi, square dancing, or regular long-duration walking, whereas the high physical activity group predominantly engaged in cycling, jogging, or swimming.

One-way ANOVA revealed no significant differences among the three groups with respect to age, height, body mass, body mass index (BMI), systolic blood pressure, diastolic blood pressure, smoking status, or alcohol consumption (all *p* > 0.05), indicating good baseline comparability across groups. Details are shown in Table 2.

**Table 2:** Basic characteristics of study participants.

Baseline characteristics	Sex	Category	Physical activity level grouping		
			Group L (*n* = 24/24/48)	Group M (*n* = 29/29/58)	Group H (*n* = 21/28/49)
Age [year]	Men	–	53.56 ± 2.65	54.19 ± 2.87	53.70 ± 3.42
	Women	–	53.96 ± 3.56	55.20 ± 2.85	53.21 ± 2.56
	Men and women combined	–	53.75 ± 3.09	54.73 ± 2.88	53.43 ± 2.96
Height [cm]	Men	–	172.76 ± 6.09	172.25 ± 5.83	175.09 ± 6.10
	Women	–	161.28 ± 6.23	161.67 ± 5.27	160.79 ± 7.21
	Men and women combined	–	167.26 ± 8.41	166.58 ± 7.65	167.24 ± 9.80
Weight [kg]	Men	–	76.77 ± 11.46	73.46 ± 9.86	75.09 ± 9.21
	Women	–	60.45 ± 8.93	60.08 ± 6.91	61.60 ± 9.27
	Men and women combined	–	68.95 ± 13.13	66.29 ± 10.71	67.69 ± 11.39
BMI [kg/m^2^]	Men	–	25.69 ± 3.18	24.73 ± 2.85	24.54 ± 2.00
	Women	–	23.20 ± 2.85	23.00 ± 2.64	23.80 ± 3.07
	Men and women combined	–	24.50 ± 3.25	23.80 ± 2.85	24.13 ± 2.64
Systolic pressure [mmHg]	Men	–	131.52 ± 10.31	131.92 ± 10.01	128.52 ± 14.62
	Women	–	127.00 ± 14.59	124.27 ± 11.38	119.82 ± 12.11
	Men and women combined	–	129.35 ± 12.62	127.82 ± 11.34	123.75 ± 13.87
Diastolic pressure [mmHg]	Men	–	79.48 ± 6.67	79.27 ± 8.20	80.22 ± 9.87
	Women	–	81.13 ± 11.04	81.70 ± 8.63	74.25 ± 7.43
	Men and women combined	–	80.27 ± 8.97	80.57 ± 8.44	76.94 ± 9.04
Smoke [*n*(%)]	Men	Deny	16(66.67)	22(78.57)	16(76.19)
		Yes	8(33.33)	6(21.43)	5(23.81)
	Women	Deny	21(87.50)	26(86.67)	25(89.29)
		Yes	3(12.50)	4(13.33)	3(10.71)
	Men and women combined	Deny	37 (77.08)	48 (82.76)	41 (83.67)
		Yes	11 (22.91)	10 (17.24)	8 (16.33)
Drink [*n*(%)]	Men	Never	3(12.50)	9(32.14)	3(14.29)
		Social drinking	15(62.50)	13(46.43)	12(57.14)
		Heavy drinking	6(25.00)	6(21.43)	6(28.57)
	Women	Never	15(62.50)	20(66.67)	24(85.71)
		Social drinking	8(33.33)	8(26.67)	3(10.71)
		Heavy drinking	1(4.17)	2(6.66)	1(3.57)
	Men and women combined	Never	18 (37.50)	29 (50.00)	27 (55.10)
		Social drinking	23 (47.91)	21 (36.20)	15 (30.61)
		Heavy drinking	7 (14.59)	8 (13.79)	7(14.29)

### 3.2 Effects of physical activity level on body composition

As shown in Table 3, in the male sample, significant differences were observed among physical activity levels in waist-to-hip ratio (WHR), body fat percentage, and visceral fat area (all *p* < 0.05), whereas no significant difference was found in muscle mass (*p* > 0.05). *Post hoc* analyses revealed a significant decreasing trend in WHR and visceral fat area across physical activity groups (L > M > H, *p* < 0.05). For body fat percentage, the low physical activity group showed significantly higher values than both the moderate and high physical activity groups (*p* < 0.05), while no significant difference was observed between the moderate and high groups.

**Table 3:** Effects of different levels of physical activity on body composition outcomes.

Sex	Physical activity level	Body composition			
		Waist-to-hip ratio (WHR)	Body fat percentage (%)	Muscle mass (kg)	Visceral fat area (cm^2^)
Men	L group (*n* = 24)	0.95 ± 0.06^a^	28.15 ± 5.83^a^	57.79 ± 10.28^a^	125.43 ± 28.77^a^
	M group (*n* = 29)	0.91 ± 0.05^b^	23.86 ± 8.08^b^	54.22 ± 7.02^a^	95.11 ± 28.02^b^
	H group (*n* = 21)	0.88 ± 0.04^c^	22.61 ± 5.82^b^	55.81 ± 11.69^a^	65.04 ± 21.09^c^
	*F*-value	13.14	4.27	0.91	29.10
	*p*-value	<0.001	0.018	0.408	<0.001
Women	L group (*n* = 24)	0.90 ± 0.06^a^	32.76 ± 5.66^a^	43.15 ± 9.27^a^	87.78 ± 38.02^a^
	M group (*n* = 29)	0.86 ± 0.04^b^	30.50 ± 5.20^a^	40.15 ± 4.23^a^	65.99 ± 22.25^b^
	H group (*n* = 28)	0.85 ± 0.04^b^	27.23 ± 7.43^b^	43.37 ± 8.10^a^	52.45 ± 22.13^b^
	*F*-value	8.98	5.29	1.66	10.52
	*P*-value	<0.001	0.007	0.196	<0.001
Men and women combined	L group (*n* = 48)	0.93 ± 0.06^a^	30.45 ± 6.14^a^	50.47 ± 12.19^a^	106.60 ± 38.40^a^
	M group (*n* = 58)	0.88 ± 0.05^b^	27.18 ± 7.52^b^	47.19 ± 9.13^a^	80.55 ± 29.06^b^
	H group (*n* = 49)	0.86 ± 0.05^c^	25.25 ± 7.11^b^	48.70 ± 11.51^a^	57.85 ± 22.37^c^
	*F*-value	19.46	6.89	1.19	31.01
	*P*-value	<0.001	0.001	0.308	<0.001

Different superscript letters (a, b, c) within the same indicator indicate statistically significant differences among physical activity groups based on the LSD post hoc test, whereas identical letters indicate no significant difference. F and p values represent the results of one-way analysis of variance (ANOVA).

In the female sample, significant differences were also detected in WHR, body fat percentage, and visceral fat area across physical activity levels (all *p* < 0.05), whereas muscle mass did not differ significantly among groups. Pairwise comparisons indicated that WHR and visceral fat area were significantly higher in the low physical activity group compared with the moderate and high groups (*p* < 0.05), with no significant difference between the moderate and high groups. Regarding body fat percentage, the high physical activity group exhibited significantly lower values than both the low and moderate groups (*p* < 0.05), whereas no significant difference was observed between the low and moderate groups.

In the combined sample of men and women, significant differences in WHR, body fat percentage, and visceral fat area were observed across physical activity levels (all *p* < 0.05), while muscle mass remained comparable among groups (*p* > 0.05). WHR and visceral fat area demonstrated a clear decreasing trend with increasing physical activity levels (L > M > H), whereas body fat percentage was significantly higher in the low physical activity group than in the moderate and high groups (*p* < 0.05), with no significant difference between the moderate and high groups.

### 3.3 Effects of physical activity level on cardiorespiratory fitness and vascular function

As shown in Table 4, significant differences in cardiorespiratory fitness and vascular function were observed across physical activity levels in both men and women aged 50–60 years; however, the patterns of change exhibited certain sex-specific characteristics.

**Table 4:** Effects of different levels of physical activity on cardiorespiratory and vascular outcomes.

Physical activity level		Cardiorespiratory and vascular function						
		Cardiorespiratory fitness			Vascular function			
Sex	Physical activity level	Absolute VO₂max	Relative VO₂max	Cardiac functional capacity (METs)	Left CAVI	Right CAVI	Left ABI	Right ABI
Men	Group L (*n* = 24)	2.25 ± 0.39^b^	30.96 ± 4.24^c^	8.63 ± 1.06^c^	7.59 ± 0.91^a^	7.63 ± 0.97^a^	0.98 ± 0.10^c^	0.98 ± 0.10^c^
	Group M (*n* = 29)	2.45 ± 0.62^b^	35.83 ± 5.49^b^	10.22 ± 1.55^b^	6.25 ± 0.79^b^	6.26 ± 0.77^b^	1.05 ± 0.09^b^	1.08 ± 0.08^b^
	Group H (*n* = 21)	3.01 ± 0.73^a^	41.86 ± 7.90^a^	11.99 ± 2.27^a^	5.84 ± 0.90^b^	5.74 ± 0.90^c^	1.11 ± 0.10^a^	1.11 ± 0.11^b^
	*F-*value	9.75	18.88	22.86	26.33	28.70	9.82	11.96
	*P*-value	<0.001	<0.001	<0.001	<0.001	<0.001	<0.001	<0.001
Women	Group L (*n* = 24)	2.30 ± 0.62^b^	34.75 ± 3.93^b^	9.37 ± 1.12^b^	7.81 ± 0.77^a^	7.83 ± 0.83^a^	0.97 ± 0.07^b^	0.98 ± 0.06^b^
	Group M (*n* = 29)	2.29 ± 0.60^b^	35.41 ± 6.33^b^	10.10 ± 1.79^b^	6.50 ± 0.87^b^	6.54 ± 0.89^b^	1.07 ± 0.10^a^	1.07 ± 0.10^a^
	Group H (*n* = 28)	3.42 ± 0.95^a^	46.68 ± 10.33^a^	13.33 ± 2.95^a^	5.74 ± 0.83^c^	5.81 ± 0.81^c^	1.06 ± 0.12^a^	1.07 ± 0.09^a^
	*F*-value	21.01	21.98	26.45	41.19	37.77	7.60	9.01
	*P-*value	<0.001	<0.001	<0.001	<0.001	<0.001	0.001	<0.001
Men and women combined	Group L (*n* = 48)	2.27 ± 0.51^b^	32.85 ± 4.47^c^	9.00 ± 1.14^c^	7.70 ± 0.84^a^	7.73 ± 0.90^a^	0.98 ± 0.08^c^	0.98 ± 0.08^c^
	Group M (*n* = 58)	2.37 ± 0.61^b^	35.62 ± 5.88^b^	10.16 ± 1.66^b^	6.37 ± 0.84^b^	6.40 ± 0.84^b^	1.06 ± 0.10^b^	1.08 ± 0.09^b^
	Group H (*n* = 49)	3.24 ± 0.88^a^	44.61 ± 9.58^a^	12.76 ± 2.74^a^	5.78 ± 0.85^c^	5.78 ± 0.84^c^	1.08 ± 0.11^b^	1.09 ± 0.10^b^
	*F-*value	30.64	38.62	47.62	66.25	65.74	15.61	20.54

Different superscript letters (a, b, c) within the same indicator indicate statistically significant differences among physical activity groups based on the LSD *post hoc* test, whereas identical letters indicate no significant difference. *F*- and *p*-values represent the results of one-way analysis of variance (ANOVA).

With respect to cardiorespiratory fitness, men demonstrated a relatively clear dose–response relationship. Relative VO₂max and cardiac functional capacity increased progressively with higher physical activity levels (H > M > L), whereas absolute VO₂max was significantly higher in the high physical activity group compared with the moderate and low groups, with no significant difference between the moderate and low groups. In contrast, among women, significant improvements in cardiorespiratory fitness were primarily observed between the high physical activity group and the other two groups, while differences between the moderate and low groups were not significant. This pattern suggests that improvements in cardiorespiratory fitness among women may depend more on higher-intensity or greater-volume physical activity stimuli.

Regarding vascular function, CAVI values decreased with increasing physical activity levels in both sexes, reflecting reduced arterial stiffness. In women, both left and right CAVI exhibited a clear graded decline (L > M > H). In men, left CAVI tended to stabilize between the moderate and high physical activity groups, whereas right CAVI showed a continuous decreasing trend. This finding suggests that vascular elasticity in women may be more responsive to variations in physical activity level. ABI results indicated that, in both men and women, individuals in the moderate and high physical activity groups had significantly higher ABI values than those in the low group, whereas no significant difference was observed between the moderate and high groups, indicating a potential plateau effect. This pattern implies that once physical activity reaches a moderate level, peripheral perfusion may already be substantially improved, with relatively limited additional benefit from further increases in activity.

Overall, higher physical activity levels were closely associated with enhanced cardiorespiratory fitness and improved arterial elasticity; however, sex-specific response patterns were evident. Men exhibited more pronounced progressive improvements in aerobic capacity indicators, whereas women showed clearer graded changes in vascular elasticity measures. These differences may be related to sex-specific physiological factors, including differences in muscle mass proportion, fat distribution, vascular structural characteristics, and hormonal regulation. Although the cross-sectional design of this study precludes causal inference, the findings underscore the importance of accounting for sex differences when developing exercise intervention strategies for middle-aged and older adults.

### 3.4 Correlations between physical activity, cardiorespiratory fitness, vascular function, and physiological parameters

**Figure 1:**
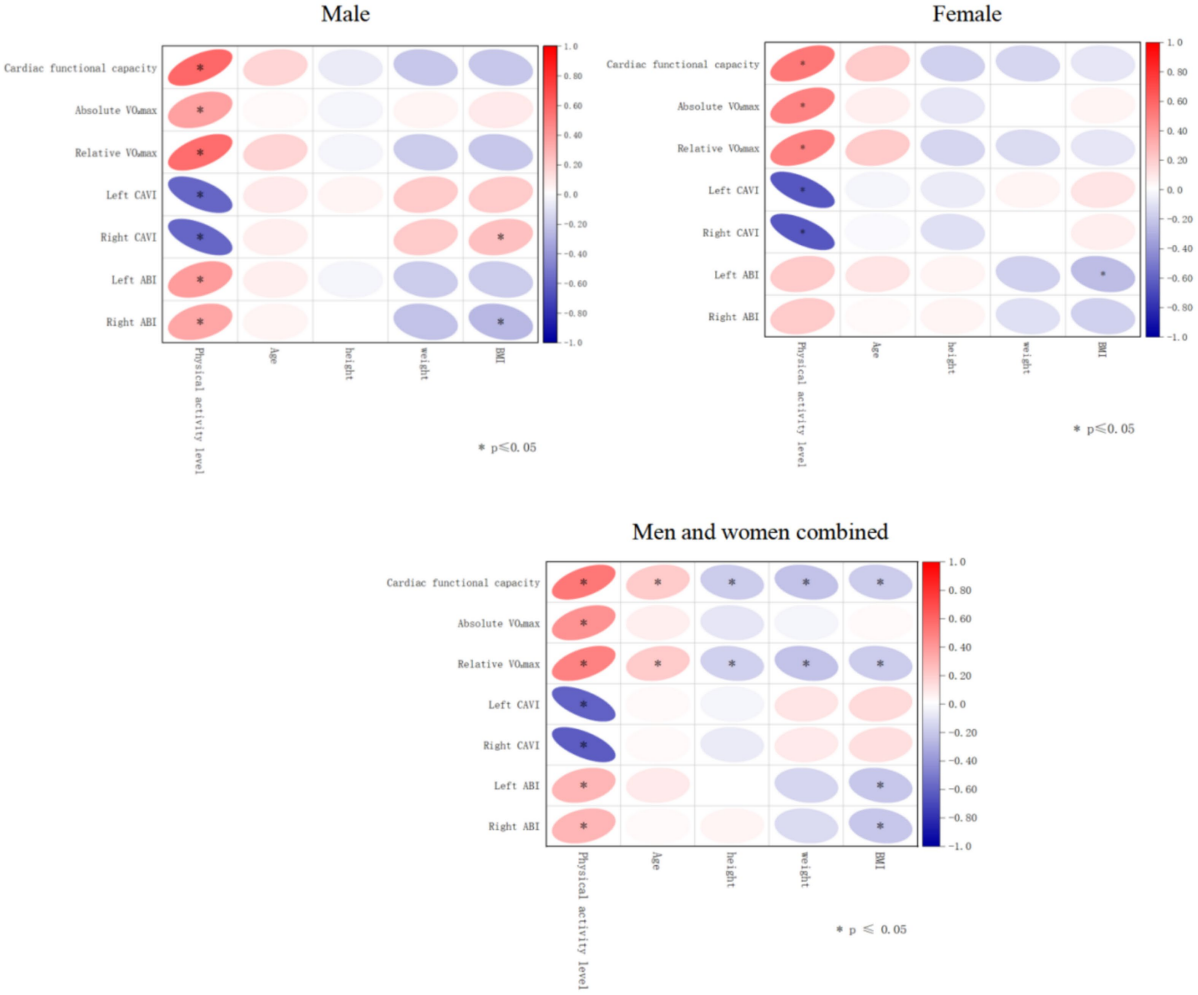
Heat map of correlation matrices between various physiological parameters and vascular function indicators.

Pearson correlation analysis revealed (Table 5 and Figure 1) that total physical activity volume (MET-min/week) was positively correlated with absolute VO₂max, relative VO₂max, cardiac functional capacity, left ABI, and right ABI (all *p* < 0.01). In contrast, physical activity volume was negatively correlated with left and right CAVI (both *p* < 0.01), indicating a favorable association between higher physical activity levels and improved vascular function.

**Table 5:** Pearson correlation analysis results of physical activity level and cardiorespiratory and vascular function.

Sex	Physical activity indicators	Absolute VO₂max	Relative VO₂max	Cardiac functional capacity	Left CAVI	Right CAVI	Left ABI	Right ABI
Men	Physical activity level (MET-min/week)	0.37**	0.56**	0.59**	−0.59**	−0.60**	0.38**	0.35**
Women	Physical activity level (MET-min/week)	0.49**	0.48**	0.53**	−0.65**	−0.65**	0.21	0.22
Men and women combined	Physical activity level (MET-min/week)	0.43**	0.51**	0.54**	−0.62**	−0.62**	0.30**	0.29**

**P < 0.01 (two-tailed).

Matrix correlation analysis further demonstrated (Figure 1) that systolic blood pressure was positively correlated with left and right CAVI and negatively correlated with left and right ABI. Diastolic blood pressure showed positive correlations with both left and right CAVI. Age was negatively correlated with VO₂max.

Sex-stratified analyses revealed modest differences in the associations between physical activity and cardiac function as well as CAVI and ABI between men and women, suggesting sex-specific response patterns.

Together, these findings suggest that physical activity plays a beneficial role in modulating cardiorespiratory fitness and vascular function among adults aged 50–60 years.

### 3.5 Moderating effect of cardiorespiratory fitness on the association between physical activity and vascular function

Pearson correlation analyses indicated that physical activity, cardiorespiratory fitness, and vascular function were all significantly correlated with each other (*p* < 0.01), suggesting close statistical associations among the three variables. However, correlation analysis reflects only linear associations and does not clarify whether the strength of the association between physical activity and vascular function varies across different levels of cardiorespiratory fitness. Therefore, a moderation model was further employed to examine the moderating role of cardiorespiratory fitness in the relationship between physical activity and vascular function.

Given that left and right CAVI and ABI exhibited highly consistent directions and statistical characteristics and are all indicators of vascular functional status, left CAVI, right CAVI, left ABI, and right ABI were standardized and summed to construct a composite vascular function index, thereby enhancing result stability and overall interpretability. Relative VO₂max, which is widely recognized as the gold-standard indicator of cardiorespiratory fitness (CRF), was selected as the representative measure of cardiorespiratory fitness for subsequent analyses.

After controlling for the main effects of physical activity and cardiorespiratory fitness, the interaction term between physical activity and cardiorespiratory fitness was entered into the regression model (Table 6). In the total sample, the moderation model demonstrated good overall fit (*R*^2^ = 0.4811, *F* = 45.7398, *p* < 0.001), explaining approximately 48.11% of the variance in vascular function. The highest-order unconditional interaction test indicated that the interaction term significantly increased the model’s explanatory power (Δ*R*^2^ = 0.0588, *F* = 16.7598, *p* < 0.001), confirming a significant moderating effect of cardiorespiratory fitness on the association between physical activity and vascular function.

**Table 6:** Overall model fit and interaction effect of the moderation regression models.

Sex	Model specification	*R* ^2^	*F-*value	*P*-value	Interaction term Δ*R*^2^	Interaction term *F*	Interaction term *P*
Men	Adjusted model	0.440	18.319	<0.001	0.107	13.345	<0.001
Women	Adjusted model	0.405	17.454	<0.001	0.054	6.924	0.010
Men and women combined	Adjusted model	0.481	45.740	<0.001	0.059	16.760	<0.001

In sex-stratified analyses, the moderation model was statistically significant in men (*R*^2^ = 0.440, *F* = 18.319, *p* < 0.001), accounting for 44.0% of the variance in vascular function. The interaction term was also significant (Δ*R*^2^ = 0.107, *F* = 13.345, *p* < 0.001), indicating a relatively strong moderating effect of cardiorespiratory fitness. In women, the overall moderation model likewise reached statistical significance (*R*^2^ = 0.405, *F* = 17.454, *p* < 0.001), explaining 40.5% of the variance. The interaction term remained significant (Δ*R*^2^ = 0.054, *F* = 6.924, *p* = 0.010), suggesting that cardiorespiratory fitness also moderated the relationship between physical activity and vascular function in women, although the incremental contribution was smaller than that observed in men.

Overall, cardiorespiratory fitness significantly moderated the association between physical activity and vascular function in both sexes. The larger increase in explained variance attributable to the interaction term in men (Δ*R*^2^ = 0.107) compared with women (Δ*R*^2^ = 0.054) suggests that the moderating effect of cardiorespiratory fitness may be more pronounced in men.

Coefficients of the Adjusted Regression Model presents the regression coefficients and corresponding statistical tests for the predictors included in the moderation regression models (Table 7). In the combined sample, physical activity was a significant positive predictor of vascular function (*β* = 0.0014, *p* < 0.001), indicating that higher physical activity levels were associated with better vascular function. Cardiorespiratory fitness also showed a significant positive association with vascular function (*β* = 0.0494, *p* < 0.001). In contrast, the interaction term between physical activity and cardiorespiratory fitness was significantly negative (*β* = −3.2 × 10^−5^, *p* < 0.001), indicating that cardiorespiratory fitness significantly moderated the association between physical activity and vascular function. This finding suggests that the strength of the association between physical activity and vascular function varied across levels of cardiorespiratory fitness.

**Table 7:** Coefficients of the adjusted regression model.

Sex	Variable	Regression coefficient (β)	SE	*T-*value	*P-*value	95% CI
Men	Constant	−0.840	0.584	−1.437	0.155	[−2.005, 0.326]
	Physical activity (X)	0.002	0.000	4.727	<0.001	[0.001, 0.002]
	Cardiorespiratory fitness (M)	0.059	0.017	3.489	<0.001	[0.025, 0.093]
	Physical activity × Cardiorespiratory fitness	0.000	0.000	−3.653	<0.001	[0.000, 0.000]
Women	Constant	−0.455	0.595	−0.765	0.447	[−1.639, 0.729]
	Physical activity (X)	0.001	0.000	3.796	<0.001	[0.001, 0.002]
	Cardiorespiratory fitness (M)	0.045	0.016	2.769	0.007	[0.013, 0.078]
	Physical activity × Cardiorespiratory fitness	0.000	0.000	−2.631	0.010	[0.000, 0.000]
Men and women combined	Constant	−0.638	0.400	−1.590	0.113	[−1.429, 0.153]
	Physical activity (X)	0.001	0.000	5.850	<0.001	[0.001, 0.002]
	Cardiorespiratory fitness (M)	0.049	0.011	4.450	<0.001	[0.028, 0.071]
	Physical activity × Cardiorespiratory fitness	0.000	0.000	−4.090	<0.001	[−0.000, −0.000]

Sex-stratified analyses demonstrated broadly consistent patterns with some differences in effect magnitude. In men, physical activity was a significant positive predictor of vascular function (*β* = 0.002, *p* < 0.001), and cardiorespiratory fitness was likewise positively associated with vascular function (*β* = 0.059, *p* < 0.001). The interaction term was statistically significant (*p* < 0.001), indicating a robust moderating effect of cardiorespiratory fitness on the relationship between physical activity and vascular function in men.

In women, physical activity also positively predicted vascular function (*β* = 0.001, *p* < 0.001), and cardiorespiratory fitness remained a significant positive predictor (*β* = 0.045, *p* = 0.007). The interaction term was significant (*p* = 0.010), indicating that cardiorespiratory fitness similarly moderated the association between physical activity and vascular function in women, although the magnitude of the interaction effect was smaller than that observed in men.

Overall, across men, women, and the combined sample, both physical activity and cardiorespiratory fitness were consistently associated with better vascular function. The significant interaction effects observed in all models further indicate that individual differences in cardiorespiratory fitness modify the extent to which physical activity is associated with vascular function.

**Figure 2:**
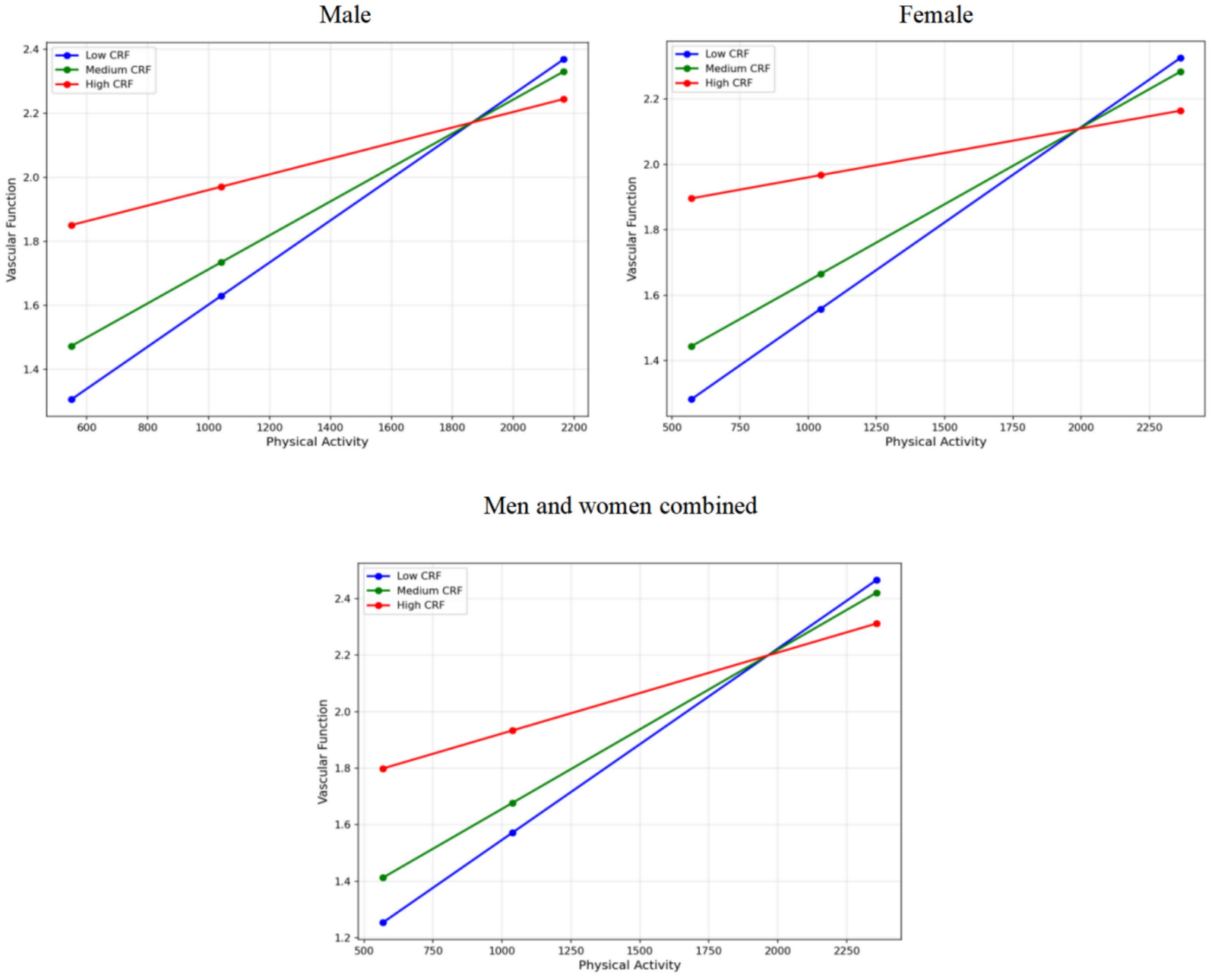
Simple slope plot illustrating the moderating effect of cardiorespiratory fitness.

Simple slope analysis was performed to further elucidate the specific pattern of the moderation effect. As illustrated in Figure 2, in the overall sample, physical activity showed the strongest positive association with vascular function at low levels of cardiorespiratory fitness (16th percentile, 30.48). At moderate levels of cardiorespiratory fitness (50th percentile, 35.00), this positive association remained statistically significant but was attenuated. At high levels of cardiorespiratory fitness (84th percentile, 46.00), the beneficial association between physical activity and vascular function was further weakened.

In the male subgroup, a conditional effect pattern similar to that observed in the overall sample was identified. Specifically, when cardiorespiratory fitness was low, physical activity exerted the strongest positive association with vascular function, whereas this association progressively diminished as cardiorespiratory fitness increased. A comparable trend was also observed in the female subgroup, with the positive predictive association between physical activity and vascular function gradually decreasing as cardiorespiratory fitness increased from low to high levels. Taken together, both in the overall sample and in sex-stratified analyses, cardiorespiratory fitness exhibited a significant negative moderating effect on the positive association between physical activity and vascular function. These findings indicate that when individuals have higher levels of cardiorespiratory fitness, the marginal vascular benefits associated with additional physical activity are relatively reduced.

Overall, the present study not only confirms the independent positive associations of physical activity and cardiorespiratory fitness with vascular function, but also demonstrates the moderating role of cardiorespiratory fitness in this relationship. Moreover, the observed sex-related differences in the magnitude of the moderation effect provide a theoretical basis for developing more precise, sex-specific exercise intervention strategies in middle-aged and older adults.

Overall, the present findings demonstrate that cardiorespiratory fitness (CRF) plays a significant moderating role in the relationship between physical activity and vascular function. As CRF increases, the positive association between physical activity and vascular function progressively attenuates and becomes non-significant at very high levels of CRF. Specifically, physical activity remains significantly and positively associated with vascular function at low, moderate, and relatively high levels of CRF; however, the strength of this association declines as CRF increases, indicating a clear pattern of diminishing marginal returns.

Sex-stratified analyses further revealed a similar moderating pattern in both men and women, whereby higher levels of CRF were associated with a weaker vascular benefit derived from physical activity. Notably, the magnitude of the moderating effect appeared greater in men, suggesting that CRF may exert a more pronounced influence on the physical activity–vascular function relationship in men individuals. This sex difference may reflect underlying variations in physiological characteristics and vascular regulatory mechanisms; however, the specific biological pathways remain to be elucidated.

In summary, although both physical activity and CRF independently represent important positive predictors of vascular function, the extent to which physical activity contributes to vascular improvement is strongly contingent on an individual’s CRF level. Among individuals with lower CRF, increases in physical activity may yield more substantial vascular benefits, whereas in those with already high CRF, additional gains associated with physical activity appear comparatively limited, consistent with a plateau-like statistical pattern.

## 4 Discussion

The present study investigated the associations among physical activity, cardiorespiratory fitness, and vascular function in adults aged 50–60 years, with a particular focus on the moderating role of cardiorespiratory fitness and potential sex differences. The main findings can be summarized as follows: (1) higher levels of physical activity were associated with better cardiorespiratory fitness and more favorable vascular function; (2) these associations exhibited clear dose–response patterns with sex-specific characteristics; and (3) cardiorespiratory fitness significantly moderated the relationship between physical activity and vascular function, with a more pronounced moderating effect observed in men.

Consistent with previous evidence, long-term engagement in regular and moderate physical activity is widely recognized as an effective strategy for improving cardiovascular health. Sustained exercise induces physiological cardiac hypertrophy, enhances myocardial contractility, and improves systemic blood flow, thereby attenuating vascular dysfunction (17). In the present study, cardiorespiratory fitness demonstrated a clear dose–response relationship with physical activity. In men, fitness improved progressively across activity levels (H > M > L), whereas in women, significant improvements were primarily observed in the high physical activity group. This finding suggests that women may require higher exercise intensity or volume to elicit substantial cardiorespiratory adaptations, potentially reflecting sex-related differences in muscle mass distribution and physiological regulation. These results are consistent with previous cross-sectional and longitudinal studies reporting inverse associations between physical activity and cardiovascular disease risk, as well as positive associations with maximal oxygen uptake and cardiac functional capacity (18–20).

Exercise-induced improvements in cardiorespiratory fitness are mediated through multiple biological pathways. Unlike pathological hypertrophy, physiological cardiac hypertrophy induced by exercise occurs without myocardial fibrosis or adverse remodeling (21). Exercise stimulates skeletal muscle to release myokines that activate the IGF-1–PI3K–Akt signaling pathway, thereby regulating myocardial autophagy and apoptosis and enhancing cardiovascular metabolic function (22, 23). In addition, exercise-induced increases in endothelial shear stress promote the release of nitric oxide and prostaglandins, improving vascular tone and facilitating adaptive vascular remodeling (24, 25).

With respect to vascular function, higher physical activity levels were associated with significantly lower CAVI values in both sexes, indicating reduced arterial stiffness. Notably, women exhibited a more distinct graded decline in CAVI across activity levels, suggesting greater vascular elasticity responsiveness to physical activity. In contrast, ABI demonstrated a plateau-like pattern: moderate and high physical activity levels were both significantly higher than the low activity group, with no further increase between moderate and high levels. This finding indicates that moderate physical activity may be sufficient to substantially improve peripheral perfusion, while additional increases in activity yield diminishing marginal benefits. Moderate-intensity exercise may optimize skeletal muscle–organ crosstalk by activating PGC-1α- and AMPK-related signaling pathways, thereby enhancing endothelial autophagy, reducing inflammation, and facilitating vascular remodeling (26–28).

Importantly, moderation analyses revealed that cardiorespiratory fitness significantly influenced the strength of the association between physical activity and vascular function, with a larger incremental explanatory contribution (Δ*R*^2^) observed in men. In both sexes, the positive association between physical activity and vascular function was strongest among individuals with lower levels of cardiorespiratory fitness and progressively attenuated as fitness increased. These findings suggest that cardiorespiratory fitness functions as a key individual-level modifier, rather than a mandatory mediating pathway, in determining the vascular benefits of physical activity.

From a practical perspective, questionnaire data indicated that participants primarily engaged in moderate-intensity aerobic activities such as square dancing and prolonged walking. Previous studies have demonstrated that approximately 8,700 steps per day are associated with a nearly 60% reduction in all-cause mortality risk, while about 7,100 steps per day are associated with a 51% reduction in cardiovascular disease risk (29, 30). These findings support moderate-intensity aerobic activity as a safe and effective exercise modality for middle-aged and older adults. For women, appropriately increasing exercise intensity may further enhance cardiorespiratory fitness, whereas for men, exercise prescriptions may be optimized according to baseline fitness levels to achieve more precise vascular benefits.

## 5 Limitations

Several limitations of the present study should be acknowledged. First, physical activity was assessed using the short form of the International Physical Activity Questionnaire (IPAQ-SF), which relies on self-reported data and is therefore susceptible to recall bias and social desirability bias. Moreover, self-reported measures may tend to overestimate actual physical activity levels. In addition, the conversion of physical activity into metabolic equivalent values and the criteria used to classify activity intensity are not fully standardized across studies, which may have affected the accuracy of physical activity categorization.

Second, the sample size was relatively limited, and all participants were recruited from a single geographic region. These factors may restrict the generalizability of the findings and reduce the statistical power of the analyses.

## 6 Conclusion

In adults aged 50–60 years, moderate-to-vigorous physical activity was significantly associated with better vascular function and higher cardiorespiratory fitness, suggesting a favorable cardiovascular health phenotype that may contribute to reduced cardiovascular disease risk. Cardiorespiratory fitness acted as a significant moderator—rather than a mandatory mediator—of the physical activity–vascular function relationship, with stronger vascular benefits observed in individuals with lower fitness levels, diminishing marginal effects at higher fitness levels, and a more pronounced moderating effect in men. These findings indicate that cardiovascular health interventions in middle-aged and older adults should integrate physical activity level, baseline cardiorespiratory fitness, and sex differences, and be implemented through structured, regular, and professionally guided exercise programs.

## Data Availability

The original contributions presented in the study are included in the article/supplementary material, further inquiries can be directed to the corresponding authors.
